# Nanostructure, Plastic Deformation, and Influence of Strain Rate Concerning Ni/Al_2_O_3_ Interface System Using a Molecular Dynamic Study (LAMMPS)

**DOI:** 10.3390/nano13040641

**Published:** 2023-02-06

**Authors:** Xueqiong Fu

**Affiliations:** Shenzhen Institute of Advanced Electronic Materials, Shenzhen Institute of Advanced Technology, Chinese Academy of Sciences, Shenzhen 518055, China; xq.fu@siat.ac.cn

**Keywords:** interface fracture, plastic deformation, molecular dynamics, metal/ceramic interface

## Abstract

The plastic deformation mechanisms of Ni/Al_2_O_3_ interface systems under tensile loading at high strain rates were investigated by the classical molecular dynamics (MD) method. A Rahman–Stillinger–Lemberg potential was used for modeling the interaction between Ni and Al atoms and between Ni and O atoms at the interface. To explore the dislocation nucleation and propagation mechanisms during interface tensile failure, two kinds of interface structures corresponding to the terminating Ni layer as buckling layer (Type I) and transition layer (Type II) were established. The fracture behaviors show a strong dependence on interface structure. For Type I interface samples, the formation of Lomer–Cottrell locks in metal causes strain hardening; for Type II interface samples, the yield strength is 40% higher than that of Type I due to more stable Ni-O bonds at the interface. At strain rates higher than $1\times 10^{9}{\;s}^{-1}$, the formation of L-C locks in metal is suppressed (Type I), and the formation of Shockley dislocations at the interface is delayed (Type II). The present work provides the direct observation of nucleation, motion, and reaction of dislocations associated with the complex interface dislocation structures of Ni/Al_2_O_3_ interfaces and can help researchers better understand the deformation mechanisms of this interface at extreme conditions.

## 1. Introduction

Thermal barrier coatings (TBCs) are widely used in various energy-producing turbines such as aeronautical and utility gas turbines to protect the metallic components of the hottest part [1,2,3,4,5]. In general, these coating systems consist of ceramic coating (usually yttria-stabilized zirconia (YSZ)) for thermal insulation, bonding coating, and metal substrate (usually nickel-based superalloys). Under an elevated temperature environment, thermally grown oxide (TGO, the main component is α-Al_2_O_3_) develops between the Ni(Al) alloy bond coat and the ZrO_2_ thermal barrier, forming a large area of Ni/Al_2_O_3_ interfaces in the coating system [6]. Although the TGO protects the underlying superalloy from oxidation and corrosion, upon thermal cycling the growth of cracks along the Ni/Al_2_O_3_ interface may eventually cause the spalling of TBCs. Therefore, the durability of TBCs is often dictated by the adhesion of the Ni/Al_2_O_3_ interface. A deeper understanding of the mechanical properties and failure behaviors of the Ni/Al_2_O_3_ interface at the atomistic level provides insight into the underlying physics of interface decohesion.

The theoretical research on the microscopic fracture of Ni/Al_2_O_3_ interface mainly focus on interface adhesion properties, such as work of adhesion and interface strength. Extensive ab initio calculations have been carried out to investigate the atomic-level tensile and shear fracture of the stoichiometric Ni/Al_2_O_3_ interfaces [7,8,9,10,11]. The calculated adhesive energies of the most stable Al-terminated O site interface (1.44 J/m^2^~1.90 J/m^2^ [7,8,9,10]) turn out to be an order of magnitude lower than the macroscopic interface fracture toughness at room temperature. This huge difference mainly comes from the plasticity induced by the crack in the Ni alloys [12]. Molecular dynamics (MD) simulation has proven to be a powerful tool for analyzing plastic deformation mechanisms during metal/ceramic interface fracture [13,14,15,16,17,18]. MD simulations on Nb/NbC and Ti/TiN interface systems showed that plastic deformation mainly occurs in the relatively softer metals [17,18]. Moreover, the plastic deformation mechanisms and fracture strengths of the metal/ceramic interfaces are strongly affected by the crystallographic orientation and interface dislocation structures, as well as strain rate, metal layer thickness, etc.

When the imposed plastic strain rate cannot be accommodated completely by dislocation slip, twinning occurs [19]. The onset of twinning is a complex phenomenon that relies on the global stress distribution and dislocation density, as well as dislocation structure. Considerable efforts have been devoted to understanding the interplay between dislocation motion and twinning. Chen et al. [20] observed direct correlation between the spall strengths and the local Shockley partial, twinning partial, and Stair-rod densities at the spall failure process of Cu/Ta multilayered microstructures under shock loading. For polycrystal nickel of commercial purity (99.5%), twining is more prevalent in surface regions, and a rapid strained sample will accommodate the new dislocations more heterogeneously at the weakest part of the microstructure, namely the grain boundaries [21]. Decreasing the grains size reduces the area of a homogeneous deformation region (or length) where slip can occur before hitting an obstacle, so larger stress was required for twinning [22]. Moreover, the dislocation density plays a critical role in determining whether a material twins. Mahajan and Boucher [23,24] reported the suppression of twinning after pre-straining for both Fe and Nb. They hypothesized that when a material is strained to a level that produces a homogenous dislocation distribution, twinning is suppressed. Florando et al. [19] also reported no evidence of twinning in the 50% cold-rolled nickel samples that show a high density of dislocations within the grains.

Compared with other metal/ceramic interfaces, the underlying atomic-level mechanisms of plastic deformation in a Ni/Al_2_O_3_ interface system is less studied. First, the Ni/Al_2_O_3_ interface has complex interface dislocation structures. Due to lattice mismatch, relative orientation, and a wide variety of defects that can form at the interface, the Ni/Al_2_O_3_ interface at equilibrium develops unique structural characteristics. Atomistic simulations showed the Ni/Al_2_O_3_ interface is reconstructed at the terminating Ni layer, and the misfit dislocation is placed in the energy-preferred second monolayer of the metal side by a (n + 1 × n + 1:n × n) mismatch plane [25]. However, experimental observation by aberration-corrected transmission electron microscopy revealed the solid Ni/Al_2_O_3_ interfaces at equilibrium develop delocalized coherency in the terminating Ni layer where the misfit dislocation is introduced [26]. This unique mechanism for misfit strain reduction contradicts most simulations in the literature. Second, the Ni/Al_2_O_3_ interface lacks appropriate interface potential. For metal/oxide interfaces, the famous potential models include the discrete classical model [27,28], the fitting model [29,30], and the iterative model [31]. However, these potentials need to assume a priori in interface structure and/or potential functional form, which limits their applications in complex Ni/Al_2_O_3_ interface structure. Based on ab initio adhesive energies, Long and Chen derived a series of Rahman–Stillinger–Lemberg potentials without any prerequisite of the function forms. These potentials have proven to be successful in investigating the interface structures and adhesive properties of Ni/Al_2_O_3_ interface [25,26] and metal/MgO interfaces [32,33].

Although the microscopic fracture behaviors of metal/ceramic interfaces have been the focus of interface research for several decades, there is, however, no understanding at present of the plastic deformation mechanisms of Ni/Al_2_O_3_ interfaces with different locations of misfit plane corresponding to different possible equilibrium interface structures. In this work, we studied the tensile fracture behaviors of two kinds of Ni/Al_2_O_3_ interfaces, corresponding to the terminating Ni layer as a buckling layer and transition layer, respectively. The plastic deformation mechanisms and dislocation evolution process were investigated numerically by the molecular dynamics (MD) method. Rahman–Stillinger–Lemberg pair potential was used to describe the Ni/Al_2_O_3_ interface interaction [34]. In particular, the role of dislocation reaction and strain rate were explored.

## 2. Models and Methods

**Figure 1:**
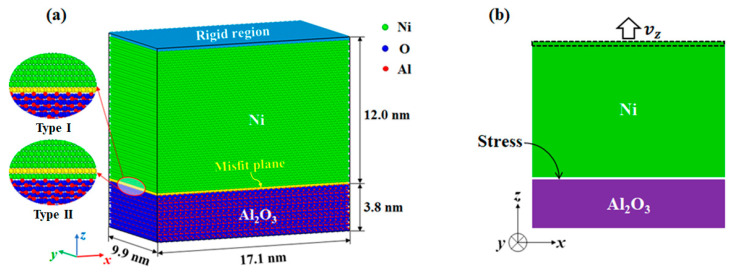
(**a**) The Ni/Al_2_O_3_ interface model showing the misfit plane located at the first monolayer and second monolayer of metal side. Type I corresponds to the first interface structure and type II corresponds to the second interface structure. (**b**) Schematic diagram of tension simulation method.

As shown in Figure 1, the most stable stoichiometric interface with Al_2_O_3_ (0001) terminated by a single Al atomic layer (*n*_Al_/*n*_O_ = 2/3) was created, and the orientation relationship was Ni$\left[\bar{2}11\right]\;$||Al_2_O_3_$\left[\bar{2}110\right]$ (*x*-axis), Ni$\left[0\bar{1}1\right]\;$||Al_2_O_3_$\left[0\bar{1}10\right]$ (*y*-axis), Ni [111] ||Al_2_O_3_[0001] (*z*-axis). Periodic boundary conditions (PBCs) were applied along *x*, *y*, and *z* directions to study the behavior of the bulk material [14,18]. To avoid the spurious interaction between top Ni atoms and bottom Al_2_O_3_ atoms, a sufficient thick vacuum region was inserted between the Ni slab and the Al_2_O_3_ slab. Considering the lattice misfit between Ni and Al_2_O_3_ (9.3%), the ratio of Ni unit cells to Al_2_O_3_ unit cells in the misfit plane was set to 11:10. According to the different atomic structures of the terminating Ni layers observed in experiment [26] and atomistic simulation [11,25], two interface models with misfit planes located at the first monolayer and second monolayer of metal side were established. For convenience, we refer to the first interface structure as Type I and the second interface structure as Type II in the following text. 

For the Ni/Al_2_O_3_ interface, the Ni-Al interaction and Ni-O interaction were described by the Rahman–Stillinger–Lemberg pair potential developed by Long and Chen [34]. The Ni-Ni interactions were described by the embedded atom method (EAM) potential developed by Mishin et al. [35]. The Al-O interactions in the Al_2_O_3_ were omitted by fixing the Al_2_O_3_ atoms in their crystal positions before interface relaxation. This is the primary approximation of the Ni/Al_2_O_3_ interface system but is sometimes reasonable for atomistic simulation studies on metal/ceramic interfaces [25,26,30,36]. First, Ni/Al_2_O_3_ interfaces are relatively brittle, and the plasticity accompanying interface cracking is due to generation and movement of dislocations within the metal [12]. Fixing the position of Al_2_O_3_ does not affect the main characteristics of equilibrium interface structure and the plastic deformation of the interface system. For example, Meltzman et al. [26] froze the Al_2_O_3_ atoms in their crystal positions and obtained the equilibrated interface by energy minimization via the conjugate gradient method. The simulation successfully reflects the periodicity of the equilibrium Ni/Al_2_O_3_ interface structure determined by aberration-corrected transmission electron microscopy. Second, the ionic interactions in the Al_2_O_3_ crystal include long-range Coulomb interactions, which significantly increase the amount of computation in molecular dynamics simulations. Fixing the position of Al_2_O_3_ can reduce the computation time and facilitate the simulation of interface models at larger scale.

The interface model was relaxed by quenching molecular dynamics [37]. In the tension simulation, the top rigid Ni atoms were subjected to a constant velocity $v_{z}$. To avoid the shock wave effect, the velocities of mobile Ni atoms were linearized from 0 to $v_{z}$ as an initial condition [38]. The strain rate $\dot{\varepsilon }$ ranged from $1\times 10^{8}$ s^−1^ to $5\times 10^{9}$ s^−1^. The simulations were performed using NVT ensemble at low temperature (1K) via a Nose–Hoover velocity scaling algorithm [39,40]. Simulations at low temperature assist understanding the governing deformation mechanisms by reducing the kinetic effects. Moreover, the results at low temperature provide insights into dislocation motion and interaction at elevated temperatures, because the effect of the temperature on the dislocation propagation is less than that on the dislocation nucleation [14]. All the simulated samples used in molecular dynamic simulations are given in Table 1. The used molecular dynamics simulation integrates Newton’s equations of motion over time to obtain the motion of the atoms/molecules in a system. MD simulations do not use the Schrödinger equation, i.e., quantic mechanics or micro-continuum media mechanics. 

The strain rate used in the molecular dynamics simulations is always several orders of magnitude higher than that used in experiments, usually less than 10^4^ s^−1^ [41,42,43]. To study the interface fracture behavior under the “low strain rate” situation, quasi-static fracture simulations under 0K were commonly used [38,44,45]. This structure minimization between each strain increment allows atoms to fully relax at each strain state. The top rigid region was successively displaced by a short distance (Δd = 0.001 Å) along the *z* axis, and after each displacement loading the mobile Ni atoms were fully relaxed under the interatomic potentials.

The interface stress was calculated as Ni-Al_2_O_3_ interaction force divided by the interface area:

(1) $$\sigma_{\alpha \beta }=\frac{1}{S}\underset{i}{\sum }\underset{j}{\sum }f_{\alpha \beta }^{\left(ij\right)}$$

where $\sigma_{\alpha \beta }$ denotes the stress at interface; α and β denote the direction of stress. $f_{\alpha \beta }^{\left(ij\right)}$ is the interaction force between nickel atom i and sapphire atom j near the interface. S is the interface area. During the tensile loading process, the interface system maintains force equilibrium, and the following four definitions of interface stress are equivalent: virial stress, interface interaction force divided by the interface area, unit area force exerted on the boundary, and derivative of total energy to the loading displacement [46].

The centro-symmetry parameter (CSP) [47] was calculated to characterize local defects (dislocation and stacking fault) and surface. The dislocation extraction algorithm (DXA) [48] was used to identify all dislocations in the plastic deformation process. We performed the MD simulations using LAMMPS [49] and visualized the atomic configurations using OVITO [50]. 

## 3. Results

### 3.1. Validation of the Potential and Interface Model

To validate the potential and interface models, we carried out tensile and shear tests to characterize the intrinsic adhesion of the coherent Ni(111)/Al_2_O_3_(0001) interface. Three interface models of the stoichiometric interface by a single Al atomic layer (*n*_Al_/*n*_O_ = 2/3) were constructed. This aluminum-terminated Al_2_O_3_(0001) surface is the ground-state configuration [51,52,53], and Ni atoms sit on O site, Al site, or hollow site. After interface relaxation, the Ni slab and Al_2_O_3_ slab were displaced along tensile direction or shear direction. For tensile test, the adhesive energy $E_{\mathrm{ad}}$ per unit area at different interface displacement $\delta_{n}$ is defined as [54]

(2) $$E_{\mathrm{ad}}\left(\delta_{n}\right)=\left[E\left(\delta_{n}\right)-E_{Ni}-E_{Al_{2}O_{3}}\right]/A$$

where $E\left(\delta_{n}\right)$ is the total energy of the interface system at interface normal displacement $\delta_{n}$ relative to equilibrium interface distance. $E_{Ni}$ is the total energy of the isolated Ni(001) layer; $E_{Al_{2}O_{3}}$ is the total energy of the isolated α-Al_2_O_3_(0001) layer; A is the interface area. 

During interface relaxation, the Ni atoms in unstable positions of the interface move along the direction parallel and/or perpendicular to the interface, causing local inelastic deformation. The generalized stacking fault energy (GSFE) of the interface was usually used to measure nonelastic deformation, especially for dislocation glide and twinning. The GSFE could be calculated by [55]

(3) $$\gamma_{SFE}\left(\delta_{t}\right)=\left[E\left(\delta_{t}\right)-E_{0}\right]/A$$

where $E\left(\delta_{t}\right)$ and $E_{0}$ are the total energy of the interface system with and without a stacking fault. A was the stacking fault area. The atomic positions perpendicular to the interface were fully relaxed, while those in the direction parallel to the interface were fixed to maintain stacking fault during interface relaxation.

**Figure 2:**
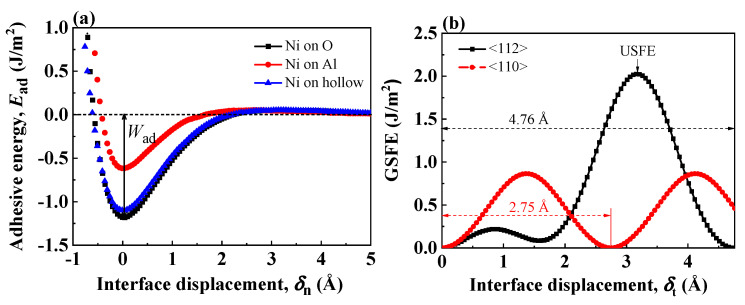
(**a**) Adhesive energy curves for the three virtual coherent Al-terminated Ni(111)/Al_2_O_3_(0001) interface structures. The well depth corresponds to the work of adhesion. (**b**) GSFE profiles of the coherent Al-terminated Ni(111)/Al_2_O_3_(0001) interface. The peak indicates the unstable SFE in the interface plane.

Figure 2a shows the adhesive energy curves under tensile test of three coherent Al-terminated Ni(111)/Al_2_O_3_(0001) interfaces with different atomic configurations. At the equilibrium interface state ($\delta_{n}=0$), the system energy as well as the adhesive energy reach the lowest. As the interface is separated to form two new free surfaces, the adhesive energy increases. The work of adhesion $W_{\mathrm{ad}}$ was defined as the energy required to break down the interfacial bonds and separate the interface into two free surfaces [56]. In Figure 2a, $W_{\mathrm{ad}}$ corresponds to the well depth of the adhesive energy curves. Figure 2b shows the GSFE profile of coherent Ni(111)/Al_2_O_3_(0001) interface along <112> and <110> crystal orientations. Considering the hexagonal symmetry of Al_2_O_3_ lattice, the shear period is 4.76 Å ($a_{Al_{2}O_{3}}$) along <112> crystal orientation and 2.75 Å along <110> crystal orientation. As the interface is sheared apart from the equilibrium state, the GSFE increases. The energy barrier along <112> crystal orientation is larger than that along <110> crystal orientation, reflecting unfavorable atomic arrangements along <112> crystal orientations [57].

Table 2 lists the work of separation $W_{\mathrm{ad}}$ obtained from tensile tests or unstable stacking fault energy (USFE) from shear tests. The atomistic simulation results are based on the Ni-O and Ni-Al interface pair potentials in the Rahman–Stillinger–Lemberg potential (RSL2) form:

(4) $$\Phi_{\mathrm{pair}}\left(r\right)=D_{0}e^{y\left(1-r/R_{0}\right)}+\frac{a_{1}}{1+e^{b_{1}\left(r-c_{1}\right)}}+\frac{a_{2}}{1+e^{b_{2}\left(r-c_{2}\right)}}+\frac{a_{3}}{1+e^{b_{3}\left(r-c_{3}\right)}}$$

where $\Phi_{\mathrm{pair}}\left(r\right)$ is $\Phi_{\mathrm{Ni}-O}\left(r\right)$ or $\Phi_{\mathrm{Ni}-\mathrm{Al}}\left(r\right)$. The potential parameters of $\Phi_{\mathrm{Ni}-O}$ and $\Phi_{\mathrm{Ni}-\mathrm{Al}}$ are listed in Ref. [34]. The work of adhesion $W_{\mathrm{ad}}$ by pair potential agree with ab initio calculation results, which proves the pair potential employed in this work gives a good description of the interface energetics for Al-terminated Ni(111)/Al_2_O_3_(0001) interface. The work of adhesion $W_{\mathrm{ad}}$ is an indication for the strength of the interface. Among the three coherent Al-terminated Ni(111)/Al_2_O_3_(0001) interfaces, the interface with Ni atom over O site shows the highest $W_{\mathrm{ad}}$, indicating this interface configuration is strong and energetically favorable. This O site preference is also quite pronounced for other metal/ceramic interfaces [16]. In fact, when the metal atoms are above the O sites, the polarization of metal atoms close to the interface is more obvious, so the interface bonding becomes stronger [34]. 

### 3.2. Interface Nanostructure

**Figure 3:**
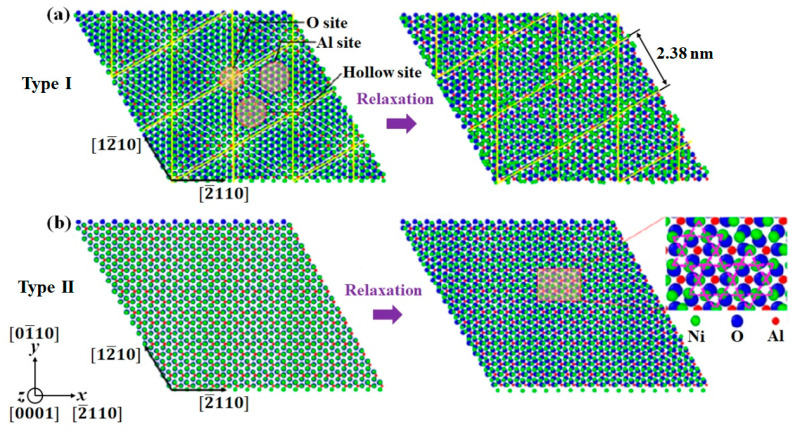
Top view of the equilibrium Ni(111)/Al_2_O_3_(0001) interface structures. (**a**) The first interface structure with misfit dislocation plane located at the first monolayer of metal side (Type I interface). (**b**) The second interface structure with misfit dislocation plane located at the second monolayer of metal side (Type II interface).

Figure 3 shows the equilibrium interface structures for both Type I and Type II Ni/Al_2_O_3_ interfaces. For Type I, the initial Ni/Al_2_O_3_ interface exhibits hexagonal symmetry, as marked by the yellow lines. After relaxation, those atoms on the Al site and O site move laterally and normally to the interface, resulting in a periodically buckled structure with a period of 2.38 nm. Concerning the atomistic structure of solid Ni-Al_2_O_3_ interfaces at equilibrium, Meltzman et al. [26] conducted TEM analysis using a monochromated and aberration-corrected TEM equipped with a post-column energy filter. The interface specimens were formed during solid-state dewetting of thin Ni film on the (0001) surface of α-Al_2_O_3_. They found similar mechanism for misfit strain reduction via a $2.5\sqrt{3}\times 2.5\sqrt{3}R30$ reconstructed interface structure. For type II interface sample, the terminating Ni layer matches perfectly with Al_2_O_3_, and Ni atoms sit on the energy preferred O site. After relaxation, the terminating Ni layer reconstructs with a combination of regular triangle and irregular hexagon structures. This reconstructed Ni layer acts as a transition layer between Ni and Al_2_O_3_ [25].

**Figure 4:**
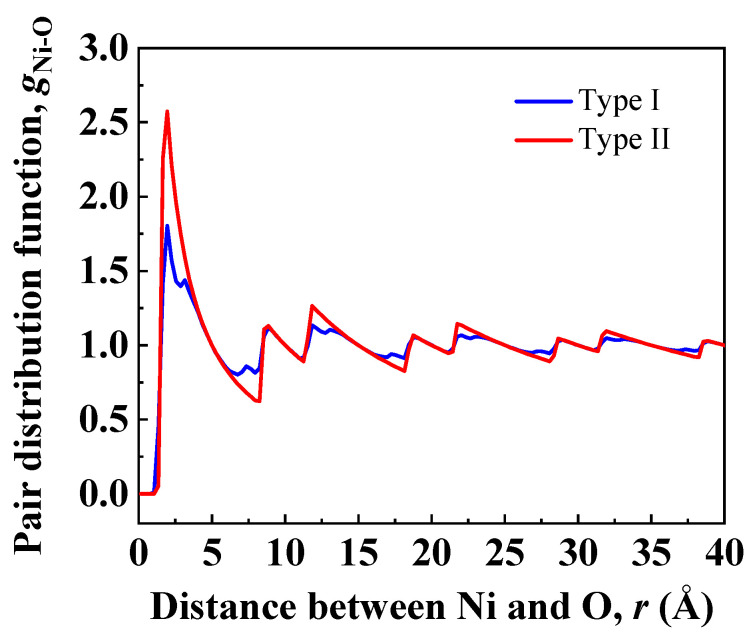
Ni-O pair distribution functions across the Ni(111)/Al_2_O_3_(0001) interfaces of Type I and Type II in Figure 3.

To investigate in detail the distribution of Ni-O bonds at interfacial regions, atomic configurations in Figure 3 were used to calculate pair distribution functions (PDF) of Ni/Al_2_O_3_ interfaces. Ni atoms in the nickel slab were picked to calculate distances to O atoms in sapphire. Figure 4 shows the Ni-O pair distribution function for particles at interfacial regions of Type I and Type II interfaces. The first peak corresponds to the first nearest neighbors, which represent Ni-O bonding neighbors. The height of the first peak of Type II interface is 64% higher than the height of the first peak of Type I interface, indicating more stable Ni-O bonds are formed at the Type II interface. Moreover, the position of the first peak of Type II interface is shifted to a lower value (1.95 Å) relative to the position of the first peak of Type I interface (2.25 Å). This suggests the Ni-O bond distance at the Type II interface is shorter than that at the Type I interface. 

### 3.3. Tensile Fracture Mechanisms

**Figure 5:**
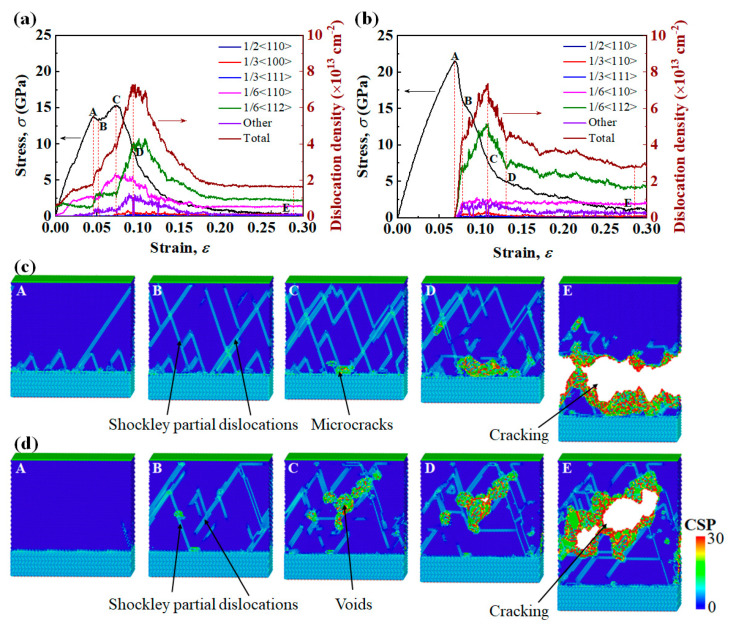
(**a**,**b**) show the simulated tensile stress–strain curves and corresponding changes of dislocation density of Ni(111)/Al_2_O_3_(0001) interface systems of Type I and Type II interface. The strain rate is 1 × 10^9^ 1/s and the temperature is 1K. (**c**,**d**) show the snapshots of the atomic microstructures of Ni(111)/Al_2_O_3_(0001) interface systems corresponding to different strains in (**a**,**b**). Atoms are colored according to the centro-symmetry parameter (CSP).

The two equilibrium interface systems were subjected to tensile loading, and the simulated interface fracture behaviors show a strong dependence on the interface structure. For Type I interface samples, Figure 5a,c show (i) a stress drop at a strain of 0.045 (point A to point B), which coincides with the nucleation of Shockley partial dislocations (1/6<112>) from the interface into the Ni layer, and (ii) strain hardening (point B to point C), which is mainly attributed to the following dislocation reaction: $\frac{\left[\bar{1}2\bar{1}\right]}{6}+\frac{\left[1\bar{1}2\right]}{6}\to \frac{\left[011\right]}{6}$. $\frac{\left[011\right]}{6}$ Lomer–Cottrell (L-C) locks effectively obstruct the movement of mobile dislocations on the slip plane. (iii) At the yield strength $\sigma_{y}$ of 15.31 GPa (point C), microcracks appear at the Ni/Al_2_O_3_ interface and stress begins to drop. As the microcracks propagate to form new free surfaces, a large quantity of dislocations escape from the free surfaces, and dislocation density begins to decrease (point D). (iv) Finally, the interface system is completely separated from the vicinity of the Ni/Al_2_O_3_ interface.

For Type II interface samples, Figure 5b,d show (i) in the initial elastic deformation stage, the stress increases linearly with increasing strain, and the yield strength $\sigma_{y}$ reaches 21.46 GPa at strain of 0.070 (point A in Figure 5b). The existence of transition layer in Type II interface increases the number of stable Ni-O bonds at the interface, so $\sigma_{y}$ of Type II interface samples is 40% higher than that of Type I interface samples. (ii) With the increase in strain, Shockley partial dislocations (1/6<112>) nucleate at the free surface of the metal layer, and the dislocation density increases significantly (point A to point B in Figure 5b). (iii) With the slipping of Shockley partial dislocations, the dislocation density saturates, and voids begin to grow within the metal (point C in Figure 5d). (iv) As strain increases further, the voids coalesce to form cracks. Finally, the interface system is separated from the middle of the metal layer (point E in Figure 5d). This fracture mode indicates that in the Ni(111)/Al_2_O_3_(0001) interface system containing the interfacial transition layer, the metal rather than the interface becomes the “weak” link.

### 3.4. Effect of Strain Rate

**Figure 6:**
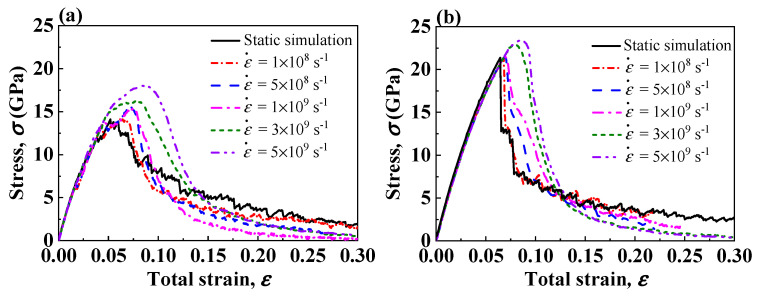
Tensile stress–strain curves of two Ni(111)/Al_2_O_3_(0001) interface models at different strain rates. (**a**) Type I interface samples. (**b**) Type II interface samples.

Figure 6a,b show the stress–strain curves for Type I and Type II interface samples under different strain rates. In the initial elastic deformation stage, the stress–strain responses are basically the same, indicating the elastic modulus of the interface system are irrelevant to the strain rate. When the deformation enters plastic stage, the stress–strain curves show strain rate dependence. The Type II interface systems show the yield strengths are insensitive to the strain rate unless the strain rate reaches $3\times 10^{9}{\;s}^{-1}$. At a higher strain rate, the dislocation segments cannot propagate fast enough to accommodate the increasing strain, and the stress rises to stimulate the dislocation propagation [58]; thus, the high strain rate in molecular dynamic simulations may cause an overestimation of the yield strength. Note that for both Type I and Type II interface samples, the interface strengths by static simulation, i.e., a quasi-static tensile test, are comparable to the interface strengths by dynamic simulation at the strain rate of $1\times 10^{8}{\;s}^{-1}$, indicating that atoms are allowed to fully relax during the tensile deformation at this strain rate. Moreover, when the strain rate is higher than $1\times 10^{9}{\;s}^{-1}$, the strain hardening phenomenon after the first stress peak in Type I interface samples disappears. At such high strain rates, dislocations cannot propagate sufficiently, and the probability of dislocation reaction decreases, so the formation of L-C locks in metal is suppressed.

**Figure 7:**
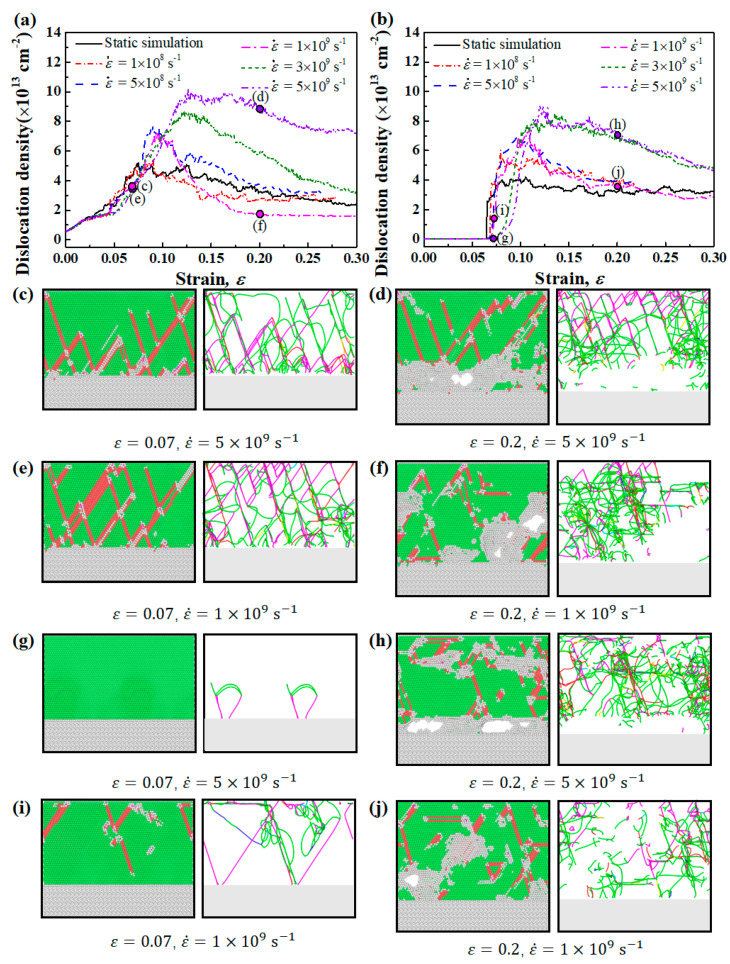
(**a**,**b**) show the simulated dislocation densities of Ni(111)/Al_2_O_3_(0001) interface systems of Type I and Type II interface. (**c**–**j**) show the snapshots of the atomic microstructures and dislocation distribution maps corresponding to the points marked in (**a**,**b**). The color-coding scheme of dislocation distribution maps in (**c**–**j**): 1/2<110> Perfect (blue), 1/6<112> Shockley (green), 1/6<110> Stair-rod (pink), 1/3<001> Hirth (yellow), 1/3<111> Frank (light blue), and Others (red).

To discover the effect of strain rate on the formation and propagation of dislocations, the dislocation density of Type I and Type II Ni(111)/Al_2_O_3_(0001) interfaces at different strain rates were computed and plotted in Figure 7a,b. The dislocation density was calculated as the total length of dislocation line in a unit volume. The corresponding microstructures and dislocation distribution maps extracted by means of DXA at strains of 0.07 and 0.2 were drawn in Figure 7c–j. For both Type I and Type II interface samples, the maximum dislocation density increases with the increase in strain rate, demonstrating that higher strain rate can promote the proliferation of dislocations. As the strain rate increases, dislocations do not have sufficient time to return to the low energy state or escape from the free surfaces formed by cracks. Therefore, the annihilation of dislocations is suppressed, and the dislocation network becomes less deformed, as shown by the more homogeneous distributed dislocation lines in Figure 7d than those in Figure 7f.

Unlike the continuous increase in dislocation density in Type I interface samples, the dislocation density in Type II interface samples increases sharply when the strain reached a critical value where Shockley partial dislocations begin to nucleate at the interface. As the strain rate increases, the critical strain at which the dislocation begins to form also increases, demonstrating that the formation of Shockley dislocations at the interface is delayed, as shown by the lower dislocation density in Figure 7g than that in Figure 7i. This restriction effect of high strain rate on dislocation emission at the interface can lead to the continuous accumulation of local stress near the interface, which is manifested as an increase in yield strength [59].

**Figure 8:**
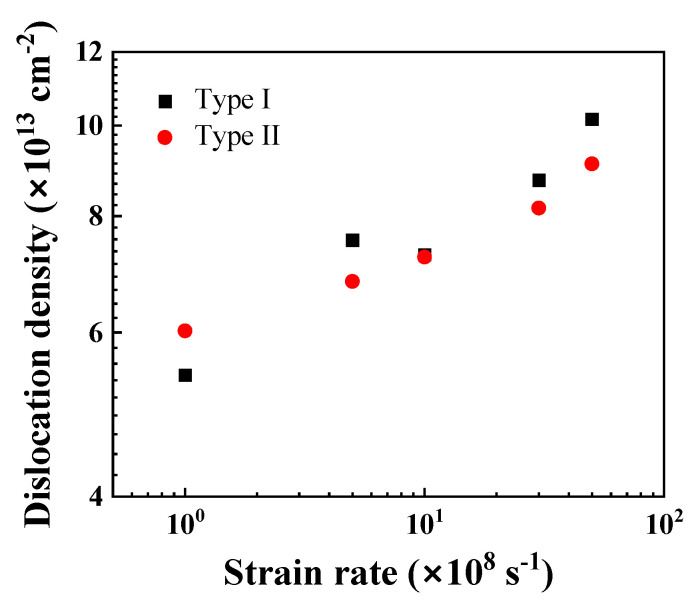
Saturated dislocation density as functions of strain rate.

Figure 8 shows the variation of the saturated dislocation density of the Ni/Al_2_O_3_ interface systems under different strain rates. For both Type I and Type II interface samples, the saturated dislocation density increases almost exponentially with the increase in strain rate, indicating rate-dependent plasticity under high strain rates [60]. Since the local re-arrangement at a Type I interface increases the number of dislocation nuclei, the dislocation density of Type I interface samples appears to be higher than that of the Type II interface samples. Note that there is no twinning phenomenon for the high value of strain rate, which differs from the twinning-induced crack kinking out of a metal–ceramics interface [61]. 

**Figure 9:**
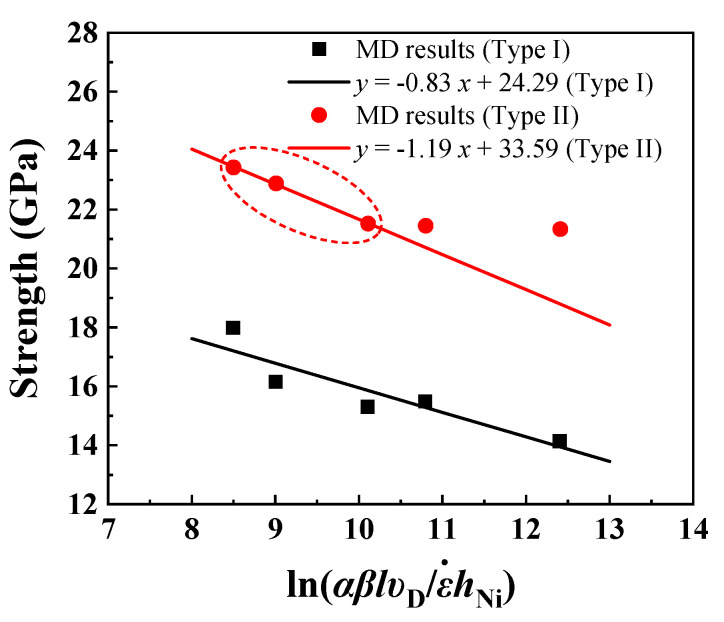
Yield strength vs. $\ln\left(\frac{\alpha \beta l\upsilon_{D}}{\dot{\varepsilon }h_{Ni}}\right)$ as $\dot{\varepsilon }$ varies for different strain rates.

At the nanometer length scale, an MD-based nucleation model has been used to predict the flow stress in nanopillars, metallic multilayers, and ceramic–metallic multilayer composites [18,62,63,64]. Based on the nucleation theory, the following constitutive equation for flow stress in nano-layers was derived [63]:

(5) $$\bar{\sigma }=\frac{Q^{*}}{S\hat{\Omega }}-\frac{k_{B}T}{S\hat{\Omega }}ln\frac{\alpha \beta l\upsilon_{D}}{\dot{\varepsilon }h_{\mathrm{Ni}}}$$

where $\frac{Q^{*}}{S\hat{\Omega }}\equiv \sigma_{athermal}$ is the athermal stress for the dislocations to nucleate. The prefactor $\frac{k_{B}T}{S\hat{\Omega }}$ has a stress unit and incorporates the effect of thermal fluctuations on the nucleation stress reduction. The activation parameters can be found by fitting Equation (5) with stress vs. strain rate results from MD simulations at a temperature of 1K as shown in Figure 9 (αβ=1, $\upsilon_{D}=1.3\times 10^{13}\;s^{-1}$, S=1, and $h_{\mathrm{Ni}}=9.21$ nm for Type I interface samples and $h_{\mathrm{Ni}}=9.15$ nm for Type II interface samples). Considering that the yield strength of Type II interface samples is sensitive to the strain rate when the strain rate is higher than $1\times 10^{9}\;s^{-1}$, MD results of Type II interface samples at strain rates of $1\times 10^{9}\;s^{-1}$, $3\times 10^{9}\;s^{-1}$, and $5\times 10^{9}\;s^{-1}$ were used for fitting. The athermal stress, thermal prefactor, and calculated activation parameters for Type I and Type II interface samples are summarized in Table 3.

At temperature as low as 1K, the effect of thermal fluctuations is negligible. The calculated nucleation stresses can be used as an approximate value of the athermal nucleation stress $\sigma_{athermal}$ of the Al-terminated Ni(111)/Al_2_O_3_(0001) interface system**.** $\sigma_{athermal}$ of type II interface samples is higher than that of type I interface samples, due to the increased effective interface area (stable Ni on O site) and stronger interface adhesion. The calculated activation volumes at 1K are two orders of magnitude lower than those computed for nucleation at room temperature from the interface in nanoscale metallic multilayers ($\hat{\Omega }=0.8b^{3}$ [63]) and ceramic-metallic multilayer composites ($\hat{\Omega }=0.5b^{3}$ [18]). Physically, the activation volume $\hat{\Omega }$ is proportional to the number of atoms involved in a thermally activated process, and $\hat{\Omega }$ decreases as the temperature decreases [65,66]. Note that α in Equation (5) is a constant that accounts for the possible emission sites and depends on the interface structure. For example, in the Type I Ni(111)/Al_2_O_3_(0001) interface, those atoms that form the periodically buckled structures are the most probable sites for dislocation nucleation. Since α is embedded in the logarithmic term, variation of α by several order of magnitude causes little change to the activation volume. Further calculations show that changing αβ from 0.4 to 1 results in a 0.08% change in the activation volume. Although an MD-based nucleation model has be used to predict the flow stress of quasistatic nanoscale experiments with strain rates at least 10 orders of magnitude lower than MD simulations [18,62], the MD results at high strain rate (10^8^/s to 10^9^/s) are usually higher than the nucleation model. This is partly because high strain rate in MD simulations may cause an overestimation of the yield strength. Moreover, the predicted yield strength by the nucleation model is usually higher compared with experiments at low strain rates since the MD simulations did not include initial defects inside the lattice such as voids and pre-existing dislocations.

## 4. Discussion

In this work, we provide the direct observation of nucleation, motion, and reaction of dislocations during tensile fracture of Ni(111)/Al_2_O_3_(0001) interfaces. The study demonstrates a correlation between interface structure, dislocation motion, and flow stress interdependencies. For Type I interface structures where the misfit plane is located at the first monolayer of metal side, the relatively large misfit between nickel and sapphire causes the interfacial Ni layers buckles in regions of weaker atomic interactions, i.e., the Ni on Al sites and Ni on hollow sites [26]. This interface reconstruction not only reduces the number of stable Ni-O bonds by nearly 40% but also increases the distance of Ni-O bonds (2.25 Å) compared with the Type II interface structure where a translation layer exists between the misfit plane and sapphire. These local re-arrangement regions in Type I interfaces become the natural sources of Shockley partial dislocations under tensile load and increase the dislocation density as well as the strength of dislocation–dislocation interactions. On the contrary, the trigonal structure of Type II interface is rearranged as a combination of a regular triangle and an irregular hexagon, and the Ni-O distance is 1.95 Å. This regular interface structure makes the Type II interface a strong link, and mobile dislocations “burst” when the strain reaches a critical value. Compared with Type I interface structure, the fracture behaviors of Type II interface structure appear to be more brittle with abrupt stress drop. It is interesting to observe that the two types of interface structures both show strong rate-dependent plasticity, though the tensile failure mechanisms differ. The saturated dislocation density increases almost exponentially with the increase in strain rate. In atomistic simulations subjected to high strain rate (1 × 10^8^ s^−1^ to 5 × 10^9^ s^−1^), crystal strength may be over-predicted [60], and interface systems yield through mechanisms that are qualitatively different from those of bulk metals. 

The methodological choices in this work were constrained by the differences between experimental conditions and simulation models in terms of model structure and loading conditions. Single crystalline models of both Ni and Al_2_O_3_ were used for simplicity. However, in thermal barrier coatings, Ni and Al_2_O_3_ are polycrystals in which grain boundary can play an important role on defect formation and hindering. Dislocation pile-up at the grain boundary makes the material harder to deform [67,68] and thus may increase interface fracture toughness. As grain sizes increase, the grain boundary acts as a source of imperfect or partial dislocations. The interaction between the propagating dislocations and the deposited and misfit dislocations may cause higher dislocation density and different hardening mechanism with the single crystalline Ni/Al_2_O_3_ interface models.

Given the complexity of the spalling process of thermal barrier coatings, the fracture of the Ni/Al_2_O_3_ interface is usually in mixed mode. This work investigated the mode I fracture to identify and quantify the major properties affecting the adhesion and fracture toughness of the Ni/Al_2_O_3_ interface. Compared with tensile failure, the interface strength corresponding to shear failure is generally lower [57], indicating less energy is required for interface shear failure. Moreover, atomistic simulations showed the shear failure of metal/ceramic interfaces was accompanied by the slip of misfit dislocation network (MDN) along the interface [46,69]. The energy barrier for MDN slip is lower than that of dislocation motion in metal. Therefore, mode I fracture was studied to investigate the plastic dissipation accompanying interface separation. 

## 5. Conclusions

The present work investigated the plastic deformation mechanisms of two kinds of Ni/Al_2_O_3_ interface systems using MD simulations. The results show that for an interface with periodic buckling of the terminating Ni layer, the formation of Lomer–Cottrell locks in metal causes strain hardening. For an interface with a regular reconstructed terminating Ni layer, a higher number of chemically stable Ni-O bonds at the interface causes higher yield strength than Model I, and the interface systems is separated from within the metal layer. Both models exhibit strain rate effects similar to those of nickel-based superalloys under high strain rates ($1\times 10^{8}{\;s}^{-1}-5\times 10^{9}{\;s}^{-1}$). The plastic deformation of metal is closely associated with the interface dislocation structures, and it influences the crack behavior and mechanical property of Ni/Al_2_O_3_ interfaces significantly. Note that there is no twinning phenomenon in the propagation of the microcracks on both Type I and Type II interfaces. Perhaps the high dislocation density (>5 × 10^17^ m^−2^) and homogenous dislocation distribution in single crystal nickel suppress the formation of twinning. This study can help researchers better understand the role of interface structure and plastic deformation on the mechanical properties of the Ni/Al_2_O_3_ interface at extreme conditions.

## Figures and Tables

**Table 1:** Simulated samples in molecular dynamic simulations.

ID	Size (Lattice)	Tensile Velocities (m/s)	Strain Rate (s^−1^)	Initial Nanostructure
S1	24 × 24 × 3 (Al_2_O_3_)-15(Ni)	1.27	1 × 10^8^	Type I
S2	24 × 24 × 3 (Al_2_O_3_)-15(Ni)	6.35	5 × 10^8^	Type I
S3	24 × 24 × 3 (Al_2_O_3_)-15(Ni)	12.70	1 × 10^9^	Type I
S4	24 × 24 × 3 (Al_2_O_3_)-15(Ni)	38.12	3 × 10^9^	Type I
S5	24 × 24 × 3 (Al_2_O_3_)-15(Ni)	63.51	5 × 10^9^	Type I
S6	24 × 24 × 3 (Al_2_O_3_)-15(Ni)	1.24	1 × 10^8^	Type II
S7	24 × 24 × 3 (Al_2_O_3_)-15(Ni)	6.20	5 × 10^8^	Type II
S8	24 × 24 × 3 (Al_2_O_3_)-15(Ni)	12.41	1 × 10^9^	Type II
S9	24 × 24 × 3 (Al_2_O_3_)-15(Ni)	37.20	3 × 10^9^	Type II
S10	24 × 24 × 3 (Al_2_O_3_)-15(Ni)	62.03	5 × 10^9^	Type II

**Table 2:** Work of adhesion $W_{\mathrm{ad}}$ and unstable stacking fault energy (USFE) for the stoichiometric Al-terminated Ni(111)/Al_2_O_3_(0001) interface.

Mode		$W_{ad}$ or USFE (J/m^2^)	
		This Work	Ab Initio
Tension	Ni on O	1.21	1.44 [9], 1.48 [10] 1.90 [34]
	Ni on Al	0.62	1.01 [34]
	Ni on hollow	1.10	1.24 [34]
Shear	<112>	1.76	1.78 [57]
	<110>	0.76	1.07 [57]

**Table 3:** Athermal stress, thermal prefactor, and calculated activation parameters for the Al-terminated Ni(111)/Al_2_O_3_(0001) interface (T = 1 K).

Interface Type	$\sigma_{athermal}$ (GPa)	$\frac{k_{B}T}{S\hat{\Omega }}$ (GPa)	$\hat{\Omega }$ b^3^	$Q^{*}\;\left(eV\right)$
Type I	24.29	0.83	0.0010	0.0025
Type II	33.59	1.19	0.0007	0.0024

## Data Availability

All data are contained within the article.
